# The Use of Photodynamic Therapy in the Treatment of Brain Tumors—A Review of the Literature

**DOI:** 10.3390/molecules27206847

**Published:** 2022-10-13

**Authors:** Dorota Bartusik-Aebisher, Aleksandra Żołyniak, Edyta Barnaś, Agnieszka Machorowska-Pieniążek, Piotr Oleś, Aleksandra Kawczyk-Krupka, David Aebisher

**Affiliations:** 1 Department of Biochemistry and General Chemistry, Medical College of The University of Rzeszów, Rzeszów University, 35-959 Rzeszów, Poland; 2 Students Biochemistry Science Club, Medical College of The University of Rzeszów, Rzeszów University, Kopisto 2a, 35-959 Rzeszów, Poland; 3 Institute of Health Sciences, Medical College of The University of Rzeszów, Rzeszów University, Kopisto 2a, 35-959 Rzeszów, Poland; 4 Department of Orthodontics, Division of Medical Sciences in Zabrze, Medical University of Silesia, 15 Poniatowskiego Street, 40-055 Katowice, Poland; 5 Center for Laser Diagnostics and Therapy, Department of Internal Medicine, Angiology and Physical Medicine, Medical University of Silesia in Katowice, 41-902 Bytom, Poland; 6 Department of Photomedicine and Physical Chemistry, Medical College of The University of Rzeszów, Rzeszów University, 35-959 Rzeszów, Poland

**Keywords:** photodynamic therapy, brain tumor, glioblastoma multiforme, 5-aminolevulinic acid

## Abstract

The treatment of neoplastic disease of the brain is still a challenge for modern medicine. Therefore, advanced methodologies are needed that can rationally and successfully contribute to the early diagnosis of primary and metastatic tumors growing within the brain. Photodynamic therapy (PDT) seems to be a valuable method of treatment for precancerous and cancerous lesions including brain tumors. The main advantage of PDT is its high efficiency, minimal invasiveness and no serious side effects, compared with chemotherapy and radiotherapy. This review was conducted through a comprehensive search of articles, scientific information databases and the websites of organizations dealing with cancer treatment. Key points from clinical trials conducted by other researchers are also discussed. The common databases such as PubMed, Google Scholar, EBSCO, Scopus, and Elsevier were used. Articles in the English language of reliable credibility were mainly analyzed. The type of publications considered included clinical and preclinical studies, systematic reviews, and case reports. Based on these collected materials, we see that scientists have already demonstrated the potential of PDT application in the field of brain tumors. Therefore, in this review, the treatment of neoplasm of the Central Nervous System (CNS) and the most common tumor, glioblastoma multiforme (GBM), have been explored. In addition, an overview of the general principles of PDT, as well as the mechanism of action of the therapy as a therapeutic platform for brain tumors, is described. The research was carried out in June 2022.

## 1. Introduction

For years, neoplastic diseases have been one of the main causes of morbidity and mortality in the world. Malignant neoplasms are classified as the second most common cause of death in Poland [1]. The most common type of brain tumor is glioblastoma multiforme (GBM), which accounts for over 70.0% of all intracranial neoplasms. Meningiomas (Latin meningioma) are also increasingly recognized. The incidence of these diseases increases with age. However, GBM is the most common Central Nervous System (CNS) tumor with an annual incidence of 3–8 cases/100,000 people [2]. According to the definition, neoplasm belongs to the group of diseases in which the cells of the body divide uncontrollably, and the new ones do not separate into common tissue cells. The loss of division control is caused by mutations in the protein-coding genes involved in the cell cycle. Mutations cause the cell to respond inappropriately to signals from the body, or the cell does not respond at all. The process of malignant neoplasm development is preceded by the occurrence of several mutations [3]. The neoplastic disease of the CNS affects the brain and spinal cord. Standard medical management includes maximum tumor resection, followed by simultaneous treatment with fractionated radiotherapy, and chemotherapy with the temozolomide prodrug [4,5]. There are many difficulties with successfully curing many CNS cancers. Commonly practiced chemotherapy or radiotherapy do not selectively affect the tumor area, which predisposes to side effects and damage to tissues not affected by the disease. Moreover, due to the anatomy of the CNS (the specific nature of the nervous tissue of the brain), many brain tumors cannot be completely removed by surgery along with the margins of the surrounding tissues (deep tumor localization/infiltration into the surrounding tissues), as traditional resection could lead to serious postoperative complications i.e., neurological disorders or disability. In such cases, there is only partial removal of the tumor or regular observation of lesions in imaging tests [6]. Many brain tumor examples exist which require the use of high-precision technology to eradicate tumors. These are often inaccessible or unsafe for treatment by traditional surgical and medical methods. Therefore, in this review we will present a site-specific treatment of Photodynamic Therapy (PDT), which has the potential to be used as a precise photochemical surgical knife to destroy tumor cells in areas that require cytotoxic control and that are inaccessible by surgical methods.

## 2. Current Brain Drug Delivery Techniques

**Figure 1:**
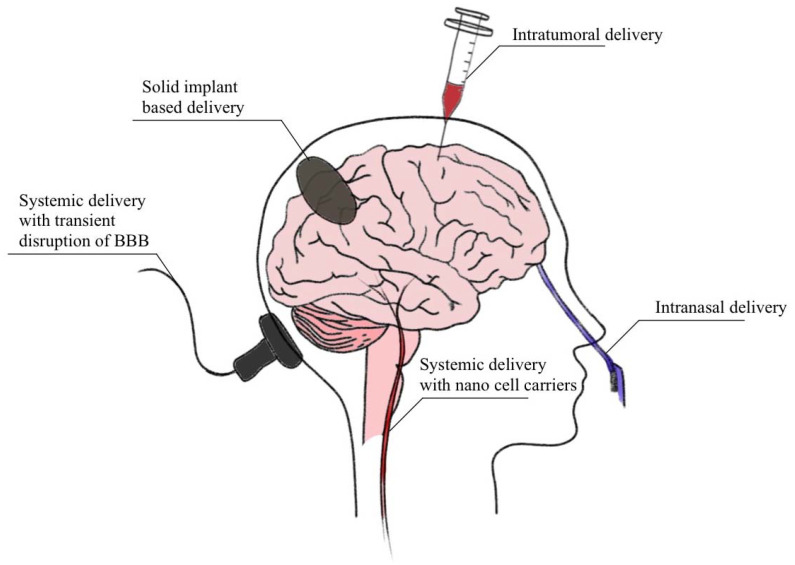
A graphic representative of current brain drug delivery techniques.

Current brain drug delivery techniques that have been clinically used are not always satisfactory. The brain is not an easily accessible anatomical site. Therefore, the objective of this review is to provide a broad overview of current strategies for brain drug delivery techniques from the past few years [7]. In conventional daily delivery, brain drugs are initially taken either orally or by injection into a vein, the subcutaneous space, or the muscle. Usually, the drug is then absorbed into local blood microcirculation or the injected site. In the next step the drug molecules enter the systemic blood circulation and are subsequently transported to various organs throughout the body. The drug molecules then pass from microcirculation to the cells and interstitial space at the brain tissue location [8]. The ideal and successful brain drug delivery should be efficient, safe, convenient to use, noninvasive and localized within a specific region. Intravenous brain drug delivery via the vascular route has obvious advantages but requires efficient drug transport across the blood–brain barrier (BBB) [9]. One of the promising solutions seems to be the ultrasound technique. Ultrasound-facilitated brain drug delivery has shown the potential to deliver drugs across the blood–brain barrier into targeted sites within the brain noninvasively [10]. Figure 1 below presents the main rounds of brain drug delivery, thus including intratumorally delivery, solid implant-based delivery, systematic delivery with transient disruption of BBB with nanotargeted carriers and intranasal or oral delivery [9,10].

**Figure 2:**
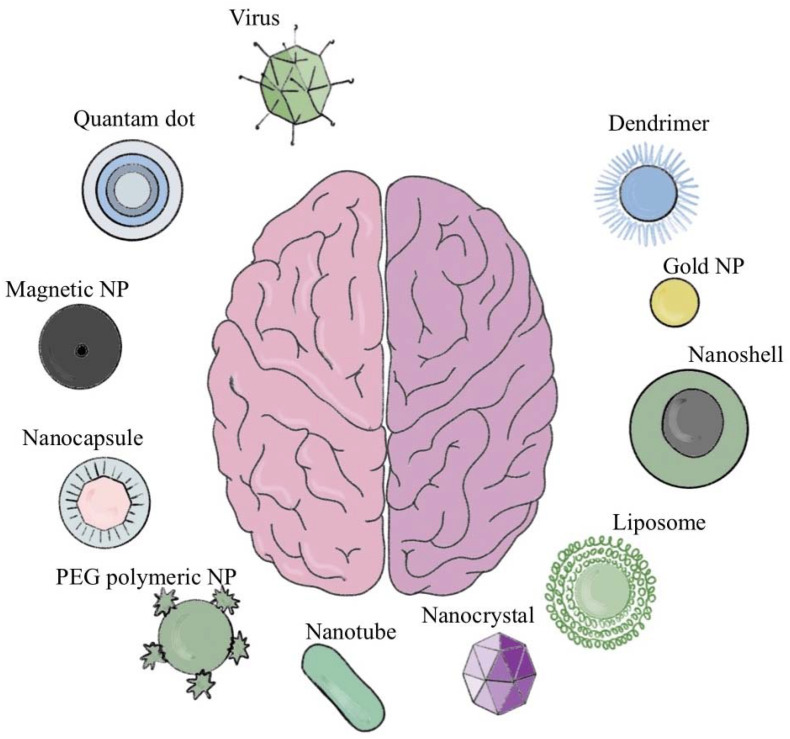
A graphical representative of the current strategies for brain drug delivery [18,19]. Abbreviation: NP-nanoparticles; PEG-polyethylenoglicol.

Current trends in brain targeting include nanotargeted agents that integrate drugs into different nanocarriers to deliver them to targeted tissues. This strategy can increase the blood-drug concentration and reduce the time of the drug delivery by improving solubility and bioavailability of hydrophobic drugs [11]. Thus, nanotargeted agents are an active area of research. However, the diagnosis and treatment of GBM remain difficult because the brain barrier restricts drug transport to the brain. Another barrier for brain drug delivery is the dynamic and heterogeneous neurological disease microenvironment. Recent advances in nanotechnology present opportunities to overcome such limitations and to deliver the drug to the brain targets with variable efficacy. Currently, available marketed nanotargeted agents for brain treatment are presented in Figure 2. To date, clinical brain tumor trials using viruses for gene delivery have employed retroviral or adenoviral vectors to introduce ganciclovir susceptibility to tumors in the form of the HSV1-TK gene. Viruses gained attention for their ability to act as vectors for therapeutic gene delivery and, as engineered infectious agents capable of selectively lysing tumor cells [12]. The advantage of Quantum dots (QDs) as nanomedicine is due to their unique optical properties that provide high sensitivity, stability and selectivity at a nanoscale range [13].

The usage of magnetic nanoparticles (MNPs) as transducers in advanced neuromodulation is promising in the field of cancer therapy. It has recently been introduced as a method for remote and wireless neuromodulation [14]. Gold nanoparticles with a core size of 2 nm covalently coated with glycans to maintain solubility, targeting molecules for brain endothelium, and cargo molecules hold great potential for delivery of therapies into the CNS [15,16]. It is estimated that the microvascular aperture of the glioma ranges from 7 to 100 nm, which is significantly smaller than peripheral tumors (380–780 nm). Therefore, small-sized molecules such as dendrimers may penetrate deeper and may obtain a more extensive tissue distribution in brain tumors than conventionally sized (approximately 100 nm) nanoparticles [17].

**Figure 3:**
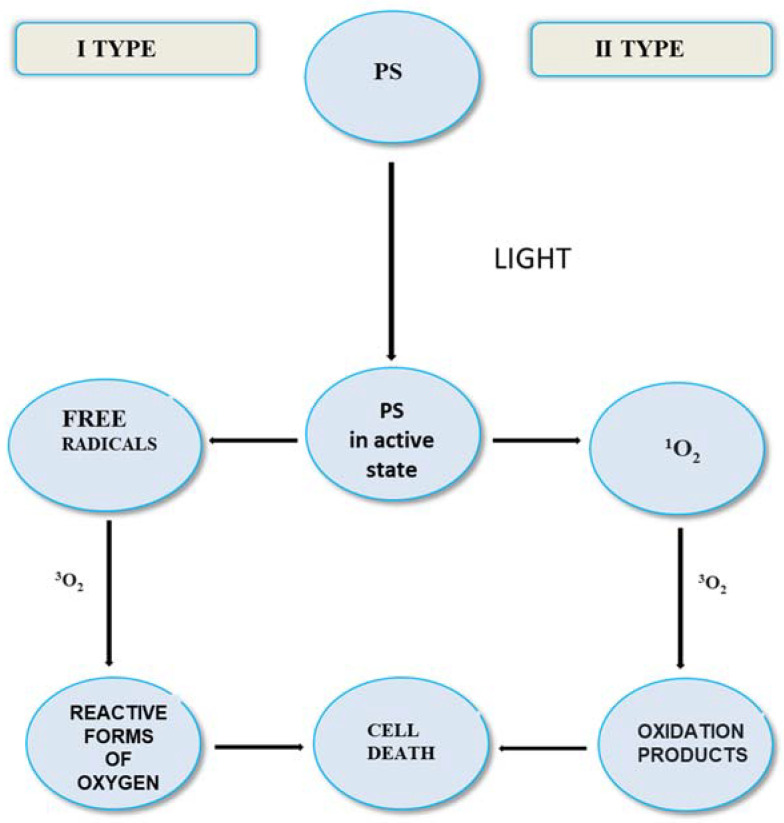
Scheme of the course of the reaction in PDT.

The current development of molecular diagnostics in medicine has significantly helped to understand the biochemistry of neoplasms [20,21]. This paper is specifically related to the advances in PDT with regard to the treatment of CNS neoplasm. PDT is a cancer treatment that uses light, a PS dye molecule and oxygen to destroy tumors. Traditionally, PDT uses intravenously injected photosensitizers to generate singlet oxygen (^1^O_2_) for the treatment of tumors. ^1^O_2_ is a critical intermediate in PDT. ^1^O_2_ can react and destroy almost any biological tissue it contacts. Figure 3 shows the course of the PDT reaction. The photodynamic reaction (RFD) is a several-step procedure. The first indispensable factor involved in it is PS. A photosensitizer is a substance that accumulates in the neoplastic tissue and selectively sensitizes it to light. Another factor is the light source with commensurate wavelength, which intensifies the PS accumulated in the tumor area. In turn, the last necessary stimulus is oxygen distributed throughout the tissue [22]. The energy obtained from the light wave is transferred to molecular oxygen and then produces ROF. The origin of RFD is the correlation between the emission bandwidth of the light source and the dye absorption bandwidth. PDT only covers tissue sites that have been treated [23,24,25]. Photosensitizers are transmitted to the human body either directly or as a result of injection [26]. The course of tumor cell degradation as a result of PDT depends on the oxygen concentration in the reaction environment. It can succumb according to two types of mechanisms. The type I reaction occurs when the susceptible PS damages cell membranes and organelles. This type of reaction is caused by free radicals that generate oxidized products. The photosensitizer can create a much more reactive form of oxygen in the triplet state (type II reaction) [27,28,29,30], therefore it is assumed that the type II mechanism is overbearing and determines the effectiveness of the therapy [31]. Both mechanisms take place at the same time, and the ratio of their share is influenced, among other factors, by the concentration of the substrate and oxygen, the pH of the environment or the dye composition [32]. It should be mentioned that only the cells that undergo PDT are closely adjacent to the site of production of the reactive oxygen species [33,34]. The effectiveness of PDT as well as its cytotoxicity is influenced by many factors, including the type of PS, the introduced dose of photosensitizer and light, as well as the presence of oxygen and the time between the introduction of PS and exposure to light [35,36].

Physicians often recommend PDT as an option for the treatment of solid tumors and macular degeneration. Many clinical examples of brain neoplasm require the use of high-precision technology to eradicate tumors that are located next to vital anatomical sites. These brain sites are often inaccessible or unsafe for treatment by traditional surgical and medical methods and include major blood vessels [37].

## 3. Brain Tumors

Brain tumors are a specific group of oncological diseases. The location of the tumor and the nature of its growth are important for clinical symptoms and the further course of treatment [38]. The Central Brain Tumor Registry of the United States (CBTRUS) has determined that both malignant and non-malignant brain tumors are numerically 10.71 per 100,000 in people 15 to 39 years old, and 40.10 per 100,000 over the age of 40 [39,40]. Glial formations are usually histologically malignant, while meningiomas are benign [41]. The etiology is not fully known. Hereditary diseases (type I and II neurofibromatosis, Hippel-Lindau disease and Li-Fraumeni disease) are postulated [42], as well as environmental and occupational factors. In addition, there are also chemical agents with a potential carcinogenic effect, including compounds formed in the process of crude oil processing, nitrosamines, various aromatic hydrocarbons, tobacco smoke, ionizing radiation, as well as previous viral infections [43,44].

The fifth edition of the WHO Classification of Tumors of the Central Nervous System (CNS) was published in 2021 [45]. This is the international standard for the classification of brain and spinal cord tumors [45].

Glioblastoma multiforme is classified as primary CNS neoplasms of glial origin. It is most common in adults [46]. Due to the highest grade of malignancy (both clinically and histopathologically), the prognosis remains poor. Patients are largely treated palliatively and die within one or two years after diagnosis [47]. In the case of GBM, only nonspecific, rare cases of distant metastases have been reported [48]. (Extracranial metastases may be present in high-grade astrocytoma, medulloblastoma and germ cell tumors) [49]. In addition, it is credited with rapid and aggressive migration, as well as increased spread in the area of the surrounding nervous tissue. It accounts for around 10.0–18.0% of all intracranial neoplasms, and 50.0–6.00% of stellate glial tumors [50,51]. The term “multiforme” is related to the conditions in which the same phenotype may arise from mutations in different subgroups of genes [52]. Its most common localization is in the frontal and temporal lobes. The clinical symptoms are related to the location of the tumor. There are general and focal symptoms. The first of these include headaches, nausea, vomiting, psycho-organic syndrome, epileptic seizures, and brain edema. The psycho-organic syndrome is also common. On the other hand, focal symptoms include various disorders, such as sensation, speech, vision, hearing, as well as paresis and cerebellar symptoms [53]. The division of the most common grades and origins of gliomas according to the WHO is presented. The increasing number of grades is associated with the increased aggressiveness of the tumor. High-grade gliomas usually affect people between 50 and 69 years of age [54]. The annual frequency of their occurrence oscillates around 5 per 100,000 people [55]. Men suffer more frequently from gliomas [56], while meningiomas largely affect women [57,58].

Two types of GBM can be distinguished—primary (pGBM) and secondary (sGBM) [59]. Primary GBM can develop rapidly, transforming into a less malignant tumor of the precursor, or it develops anew (de novo in Latin) and causes neurological symptoms. Absorption of drugs through the blood-brain route is difficult. The problem is choosing the right dose of chemotherapy, which can cause toxic reactions. On the other hand, radiotherapy causes many side effects, including the phenomenon of radio resistance [60,61,62].

According to the literature, there are two concepts regarding the formation of the most aggressive brain tumor. The first theory is that gliomas arise from astrocytes and oligodendrocytes (glial cells), which then undergo mutations in tumor genes and tumor anti-oncogens, resulting in cell differentiation and tumor formation. On the other hand, the second view suggests that the neoplasm originates from progenitor cells undergoing oncogenic transmutation and thus initiates the neoplastic process [63].

## 4. Advantages and Disadvantages of PDT Treatment

GBM is the most common malignant brain neoplasm and displays a histologic similarity to astrocytic cells [63]. Treatment of GBM currently involves tumor resection followed by concomitant radiation therapy and chemotherapy with temozolonide. Unfortunately, recurrence is nearly universal [64]. One reason for the poor prognosis of patients with GBM is that glioblastoma cells have been detected at a distance up to 4 cm beyond the identifiable borders of the tumor and it follows that 80% of recurrent GBM is found adjacent to the resection borders [64]. To improve outcomes, intraoperational visualization techniques such as intraoperative MRI and fluorescence imaging with 5-aminolevulinic acid (5-ALA) have been successfully employed; 5-ALA fluorescence imaging is currently standard use for resection of high-grade glioma in an increasing number of hospitals, and it is also increasingly used for low-grade glioma [65]. FDA approval of 5-ALA for fluorescence guided resection has also spurred a renewed interest in the application of photodynamic therapy (PDT) for treatment of GBM [64]. Since there is evidence to suggest that recurrent tumors develop from residual glioblastoma, third-generation photosensitizers (PS) for PDT that can target areas difficult to reach by microinvasion, while preserving sensitive healthy brain tissue, may improve patient outcomes [65]. Alternative therapies for treatment of glioblastoma multiforme are currently being sought as post-treatment recurrence is nearly universal with a poor prognosis. The high rate of recurrence is due to both the invasive nature of glioblastoma and its cellular resistance to traditional methods of apoptosis-inducing chemotherapy. We seek to explore the properties and potential of reagents that combine paraptosis-induction with apoptotic/necrotic photodynamic action for targeting and killing glioblastoma cells that survive standard treatments, due to either their distance from the resection margin and/or their genetic resistance to chemotherapy. The formation of singlet oxygen at a specific biological site is extremely important in order to understand the properties of tumor destruction by directed and concentrated singlet oxygen. Reactive products formed by interaction with singlet oxygen give rise to the desired toxic effect. Since singlet oxygen diffusion over a distance is unlikely, we hypothesize that specific/controlled accumulation of a sensitizer in a tumor may result from cleavage from a fiber probe. The consensus in the photodynamic therapy literature is that singlet oxygen diffusion in cells is shorter than the diameter of a typical intracellular organelle [66]. Recent papers by Niedre et al. [67,68], Kanofsky, [69,70] and Ogilby et al. [71,72] detect singlet oxygen luminescence in cells and tissues. Human gliomas are primary neoplasms of the central nervous system that grow diffusely, show different grades of local aggressiveness, and display morphologic and molecular phenotypes of glial lineages but also of less differentiated neural progenitors and stem cells [73]. Although the exact cellular origin of gliomas remains unclear [74], it has been proposed that a small fraction of cancer cells constitutes a unique reservoir of glioma initiating cells controlling tumor growth.

The focus of this review is on targeted cancer therapy. Brain cancer drug delivery is no longer simple, and it is necessary to deliver drugs in new formulations. Therefore, gene therapy can be defined as the deliberate transfer of DNA for therapeutic purposes. Our interest is in the use of lasers for nonviral targeted gene transfer. For example, Shirahata et al. made a small hole in a cell membrane by pulse laser irradiation to help a gene contained in a medium to be transferred into the cytoplasm through the hole. This hole disappears immediately with the application of laser irradiation of the appropriate power [75]. Several techniques are currently used to transfer genes into various cells, tissues and organs. Although gene therapy is a potential therapeutic approach for arterial restenosis and angiogenesis, the efficiency of transfection is low regardless of the technique used. To transfer a gene efficiently into glioma cells, a novel laser method [76] on gene transfection has been studied. However, there are therapies that can be used for this such as PDT, which is comprehensive, highly effective and a minimally-invasive photodynamic therapy. The American Food and Drug Administration (FDA) recognized PDT as a reliable method of cancer treatment. It is also used in the treatment of precancerous conditions, and it is associated with a low risk of side effects. It requires the presence of a photosensitizer (PS), oxygen and light (usually a laser-diode) of a specific wavelength. When triggered by oxygen, PS activates reactive oxygen species (ROS/ROF), i.e., singlet molecular oxygen, hydroxyl radicals and/or superoxide anions, which can cause phototoxicity or tissue damage due to their oxidation proteins, fats, or deoxyribonucleic acid (DNA). The resulting oxidation mechanisms cause damage to cell membranes (as a result of the mechanisms of apoptosis, nephrosis or autophagy) [77], leading to permanent damage and destruction of the treated tumor cells. Not only does PDT directly affect cancer cells, but it also destroys and reduces the vascularity of the tumor, causing an inflammatory response that stimulates removal of dead cells, restoration of adequate tissue homeostasis, and even systemic immunity. The discussed method of therapy does not affect the extracellular matrix (ECM); therefore, the process of tissue fusion carries a minimal risk of scarring and the risk of possible infection [77]. Current limitations of PDT include tissue irradiation with visible wavelengths that have a short tissue penetration depth. Limitations may be addressed in the design of PDT systems that can fluoresce visible light in deep tissue. Complementary research must also address facets of PDT that are diagnostic in nature, such as imaging PS delivery and measuring the amount of singlet oxygen produced on or within a cell, and the number of ^1^O_2_ molecules required to kill a single cell. PDT is a subject of intense research although few laboratory PDT systems have made the transition to clinical use. In the USA, several PS have been approved by the Food and Drug Administration and they are in use with many currently in clinical trials [78].

Injected sensitizer-oriented photodynamic therapy has been used with success for over two decades, but it is subject to fundamental limitations.

Disadvantages of current PDT:

- “Free” photosensitizer from injection must be cleared from the body;
- Better selectivity needed of injected sensitizer for diseased cells and tissue;
- Problem of skin photosensitivity from injected sensitizer;
- Higher concentrations of ^1^O_2_ are needed at target sites;
- Hypoxic tumors are inherently difficult to treat with PDT, due to the oxygen requirement for their photodestruction.

Advantages of the PDT Method:

- Far less “free” photosensitizer in body since it will be cleaved on-site by the fiber;
- Oxygen passage through fiber solves problem of hypoxia for tumor destruction;
- High precision eradication of tumors in diseased tissue adjacent to vital tissue;
- Existing endoscopic and micro-optic methods can be adapted to the new fiber device;
- Fiber method is less invasive, systemic administration of sensitizer not required;
- Newly acquired mechanistic understanding in our lab can be applied to increase singlet oxygen generation at water-fiber cap interfaces;
- Fiber system can better achieve sensitizer-O_2_ concentrations at a specific site concurrent with high excitation intensity to enhance local ^1^O_2_ concentrations.

The time is ripe for an integrative approach and the development of a hybrid photosensitizer/fiber optic device using techniques developed in diverse fields: organic synthesis, photochemistry, photobiology, and device development. Upconversion nanoparticles (UCNPs) are a unique class of optical nanomaterials doped with lanthanide ions featuring a wealth of electronic transitions within the 4*f* electron shells. These nanoparticles can upconvert two or more lower-energy photons into one high-energy photon. Recently, many examples of UCNPs with covalently attached PS have been designed. A controlled drug release is highly important for biomedical applications with potential applications of UCNPs to make them significant in research due to their unique fluorescence and luminescence properties [79]. The colloidal chemistry and interactions played important role to self-organize the UCNPs into a super lattice array within the colloidal solution. The formation of nano clusters was also identified during the self-assembly arrangement. These clusters are made with few monodispersed particles in a fascinating manner and it resulted into a periodic array of the particles to form the superlattices. The stability of the SL colloidal solutions can only be possible due to the presence of electrostatic interactions between the particles themselves As the nanoparticles are covered with ligands (oleic acid), they are dispersed transparently in a solvent (cyclohexane). UCNPs can precisely convert the long wavelength of light to ultraviolet/visible (UV/Vis) light in gas therapy for the controlled gas release owing to their unique upconversion luminescence (UCL) ability. The gases NO, O_2_, H_2_, H_2_S, SO_2_, and CO play an essential role in the physiological and pathological processes. The UCNP-based gas therapy holds great promise in cancer therapy, bacterial therapy, anti-inflammation, neuromodulation, and so on [80].

## 5. Development of Photosensitizer and Fiber Optic Technology

Tumor cells are often “hypoxic”, which presents a problem since ground-state triplet O_2_ is needed for photodynamic therapy (Equations (1) and (2)). Singlet oxygen is generated by sensitization due to the low energy of this species, sensitizers such as porphyrins are used due to their high absorptivities.

Sens + visible light (hν) → Sens* (1)

Sens* + ^3^O_2_ → Sens + ^1^O_2_ (2)

Flowing O_2_ within the optical fiber to hypoxic tumors will enable greater ^1^O_2_ uptake and enhanced photooxidative damage of the tumor. The proposed heterogeneous (fiber optic) technique aims to control the concentration of O_2_ in the medium. By achieving high local concentrations of sensitizer and O_2_, concurrent with high excitation intensities, the new fiber optic approach will minimize damage to surrounding tissue. Ground-state O_2_ is mildly toxic itself and saturating the milieu beyond a tumor site is not desired.

A fiber optic-based singlet oxygen generator for targeted singlet oxygen delivery is proposed for use in photodynamic therapy and drug delivery. The objective is to design a heterogeneous photodynamic therapy device that uses the optical excitation of sensitizer molecules released from porous ends on hollow photonic band-gap optical fibers through which O_2_ flows. The work focuses on developing the synthetic methodology to bind porous silica to the end of commercially available hollow photonic band gap optical fibers, optimize optical coupling between the fiber and bound photosensitizer, maintain porosity throughout the bound silica, and release photosensitizers from the silica matrix by visible light irradiation. A potential problem lies in selecting the appropriate solid support for the sensitizer [81]. Measuring the O_2_ flow through the xerogel capped fibers is straightforward [82].

## 6. Photosensitizers

Photosensitizers are one of the three main components of PDT. Properly selected PS should meet a number of factors, including no systemic toxicity, accumulation in tissues affected by lesions, and activation at wavelengths of light sufficient for deep penetration of the brain tissue [83]. Based on the literature reports, there are three generations of photosensitizing compounds [84,85]. First-generation PS molecules are composed of naturally occurring porphyrins, including hematoporphyrin. These compounds show an absorption of around 400 nm, while they show a limited absorption of excitation at longer wavelengths of light [23]. The first generation includes a derivative of hematoporphyrin (HpD), which is an inefficient producer of singlet oxygen and requires prolonged photostimulation [86]. The second-generation photosensitizers include chlorides (sodium talaporphine and temoporfin—used in the treatment of dermatological diseases) and 5-aminolevulinic acid, which are usually activated with wavelengths > 600 nm. Moreover, they are more effective in the production of singlet oxygen [7]. Currently, the main precursor used in the treatment of gliomas is 5-ALA [87]. Third-generation photosensitizers show greater selectivity of neoplastic cells, achieved by conjugating modifiers, including nanoparticles and antibodies [86]. The purpose of their efficient design is to reduce non-target effects while optimizing pharmacokinetic properties, as well as excitation absorption to maximize effective PDT windows while minimizing consequences [88]. Other photosensitizers or their precursors that have been used clinically in the treatment of brain tumors including HGG, include porphimer sodium (Photofrin), temoporfin and verteporfin [89]. Borated porphyrins (BOPPs) are especially useful in cerebral therapy. In combination with photodynamic therapy and the method of neutron capture, boron may predispose to the effective treatment of brain tumors [90].

### 5-aminolevulinic Acid (Pro-Drug)

**Figure 4:**
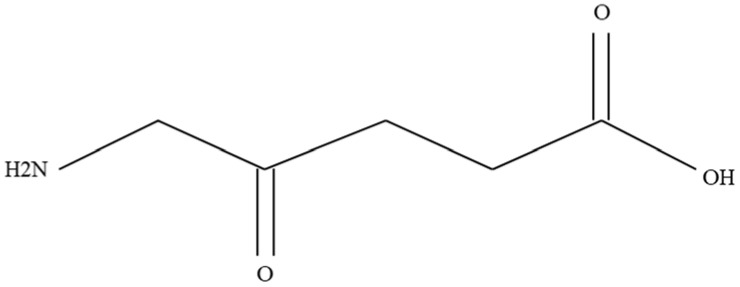
5-aminolevulinic acid molecule [104].

**Figure 5:**
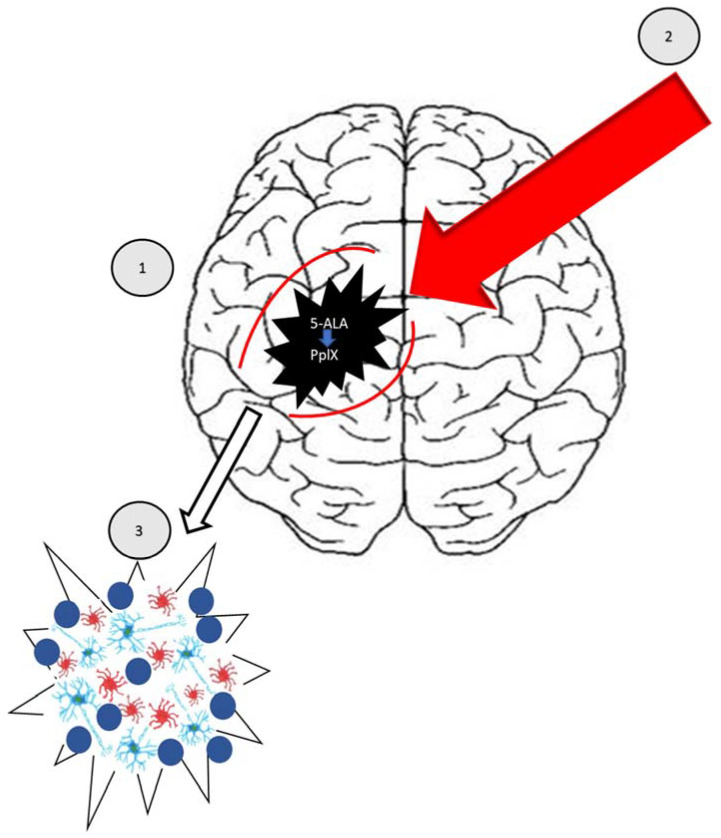
PDT in brain tumor degradation (source: own elaboration).

5-aminolevulinic acid (Figure 4) is a derivative of levulinic acid and belongs to the group of keto acids and amino acids. It is considered a precursor in the heme biosynthetic pathway. According to the available scientific literature, PDT is highly effective in combating HGG [91]. It is the only intraoperative measure that can be used during the surgical resection of gliomas under the control of fluorescence. The molecule of 5-ALA acid is shown in Figure 4. As a result of its consumption before therapy, it can accumulate in malignant brain cells and infiltrate neoplastic cells, outside the area covered by the tumor, through a process caused by increased permeability of blood vessels [92,93,94]. In the mitochondria, this acid is transformed into the organic compound Protoporphyrin IX (PpIX) and, as a result of the enzyme ferrochelatase, heme is produced. Lower expression of ferrochelatase in the diseased area may predispose to the accumulation of PpIX in brain tumors, and also lead to its predilection in the tumor [95]. Moreover, the enzyme porphobilinogen deaminase (PBGD) may also be selectable in the production of PpIX through its role in catalyzing PpIX biosynthesis [96]. In a further step, red fluorescence is released again at a wavelength of 635 nm after excitation with light near the Soret band (also known as B or y band) (around 410 nm) [97]. The use of 5-ALA/PpIX was reflected in the treatment of HHGG as a result of both FGS (with blue light activation) and PDT (with red light activation due to better tissue penetration) (Figure 5) [98,99]. As a result of a study confirming greater tumor control as well as progression free survival (PFS) with 5-ALA FGS in relation to microsurgery, the European Union approved 5-ALA for FGS HGG [100,101]. In addition, the FDA has approved 5-ALA as the first-ever fluorescent agent intended for better visualization (high sensitivity, specificity) of neoplastic tissue during surgical resection of a brain tumor [102]. Research on the improvement of PDT using ALA is still ongoing [103].

## 7. A Review of the Literature

Recent years of research by scientists have resulted in PDT being consolidated as a non-life threatening, safe method for oncology patients. Despite this, the therapy requires solid confirmation by clinical trials. The mechanisms of the method can be understood through in vitro tests on cell lines as well as in vivo tests in animals. Researchers are looking for solutions to demonstrate the effectiveness of PDT in the treatment of various malignant neoplasms, including brain tumors [105,106].

The observations showed that patients who underwent surgery combined with photodynamic therapy showed a longer survival period than those who underwent surgery only [105].

A scientific publication was published for the first time 26 years ago on the removal of high-quality gliomas with the use of 5-ALA, which contributed to its spread [106].

5-ALA acid was used during the intraoperative imaging of brain tumors. Due to the visual disclosure of undetectable tumor remnants, neoplastic lesions were completely resected, which in turn prolonged the life of the patients. The ALA-PDD method was also used at the Institute of Neurosurgery in Moscow, during the removal of malignant brain tumors in 17 patients. Tumors were subjected to microsurgical resection, by endoscopic control of the operating field, based on traditional observation in white light, and fluorescence after irradiation with blue light with a wavelength of 400 nm. It turned out that discernible fluorescence was observed in all glioblastomas. Clear observation of the operating field made it possible to completely excise the tumors [104].

The article by Stummer et al. describes a case of a patient with left frontal glioblastoma multiforme who was treated surgically with both radiotherapy and chemotherapy. One year after resection of the tumor, another lesion appeared in the left islet of the brain. Photodynamic therapy was decided upon as a result of unsuccessful attempts to remove the lesion. After oral administration of 5-ALA, irradiation was made with a diode laser. After about 24 h, the tumor was practically completely extinct in the woman, and the disease did not return after less than 5 years [105,106].

Schwartz C. et al. in their study described the case of 15 patients with inoperable gliomas (less than 4 cm) who underwent PDT and 5-ALA. Subsequently, the patients were compared with those that had glioblastoma with a diameter of 12 cm, who underwent only surgery. Patients using PDT showed a longer median progression-free survival, amounting to 16.0, while in the second group it was 10.2 months. Six patients in the PDT group lived without progression> 30 months [107].

The Royal Melbourne Hospital team has had the most clinical experience with PDT, having studied more than 350 patients with brain gliomas. The overall survival of patients with newly diagnosed and recurrent gliomas, corresponding to 28.0 and 40.0% after 2 years, and 22.0 and 34.0% after 5 years, was determined, which indicates an increase in the effectiveness of the method in relation to previous data. A similar correlation was demonstrated in over 1000 other examined patients who were included in the PDT observational studies with HGG [108,109].

In a series of 365 PDT applications with the use of 5-ALA and sodium porphimer in 150 patients with brain cancer, side effects were present in 4.7% of people. Brain edema after PDT mediated by sodium porphimer was found in 1.3% of patients with recurrent tumors [108,109].

### Cellular View

The U87-MG cell line is derived from a malignant astrocytoma grade III human tumor [110]. According to current histopathological classification of WHO, grades III and IV are the most aggressive type of malignant astrocytoma [111]. Thus, the cell line U87-MG reflects a clinical malignant astrocytic tumor. The cell line U87-MG is commonly used for neuro-oncological research and has been implemented in numerous in vitro trials regarding novel treatment modalities, including evaluation of oncotoxic chemotherapeutic and phototherapeutic agents [112]. However, the loss of differentiation of U87-MG cells is not complete, since they do express GFAP protein, albeit at a very low level in basal culture conditions. This property enables potential biomolecular analysis of the viability of the cells via comparing the GFAP expression level in different experimental groups, and it can be enhanced several fold with an antisense construct for the proto-oncogene EGFR if needed for more detailed analysis of this marker [113]. The U87-MG cell lines express high levels of peroxisome proliferator-activated receptor gamma (PPAR gamma) involved in the control of cell proliferation and apoptosis [114]. This means that these cells may react with apoptotic activation on treatment with different oncotoxic compounds. For example, it has been demonstrated that exposition on PPAR gamma agonists of the thiazolidinedione class triggers apoptosis in the human cell lines U87-MG, in contrast to primary astrocytes which are unaffected [115]. This suggests that profound differences in energy metabolism exist at the mitochondria level between glioma cell lines and primary astrocytes, and this gap may be utilized in defining the intensity of exposition as discriminator crucial for therapeutic window [116]. Since one pathway by which Ophiobolin A-porphyrin conjugates exert their cytotoxic function is as a generator of reactive oxygen species, it is crucial that the differences in mitochondrial function, including free radical turnover and scavenging between the target (glioma) cells and their physiological background, exist [117].

## 8. Conclusions

Due to the high selectivity of action, photodynamic therapy is very promising compared with classic treatments used in the field of oncology.

Despite the limitations of the sample size and several randomized controlled PDT studies, the data suggest a potential beneficial effect of PDT on a higher survival rate in patients with glioblastoma compared to standard therapy.

The main advantage of the PDT method is its high efficiency and minimally invasive character. Depending on the location of the disease, it ranges from 90% to 97% and is a very good alternative to surgery and radiotherapy. Moreover, the use of this therapy shows the possibility of generating fewer side effects than the use of chemotherapy. The recovery period is faster. Comparing photodynamic therapy to classical surgery, preoperative anesthesia of the patient is not required. There are also many contraindications to the procedure. The method also allows for the treatment of multiple disease foci. The disadvantage of PDT is its high cost. Scientific evidence confirms that photodynamic therapy is effective not only in the field of oncology but also in many other areas of medicine where surgical intervention is difficult, impossible or carries a high risk of side effects.

It is expected that the coming years will be a period of observation of the dynamic development of diagnostics and therapy with the use of 5-ALA acid. The effectiveness of PDT depends largely on professionals, scientists and researchers in the medical field, who specialize in the various stages of conducting and supervising PDT. In addition, this therapy deserves greater capacity and investment in clinical trials, which would allow scientists to draw more robust conclusions about the true translational potential of PDT.

## Data Availability

Not applicable.
